# Gastric duplication cyst in an adult: a rare case presentation and literature review

**DOI:** 10.1093/jscr/rjaf651

**Published:** 2025-08-26

**Authors:** Seng Pei Khaw, Antonio T S Tan, Martin T W Kueh, Alexander Z Y Lim, Pei Pei Lee, Aizat Aziz, Wei Keat Ooi

**Affiliations:** Department of Surgery, Royal College of Surgeons in Ireland and University College Dublin Malaysia Campus, 10450 George Town, Malaysia; Department of Surgery, Royal College of Surgeons in Ireland and University College Dublin Malaysia Campus, 10450 George Town, Malaysia; Department of Surgery, Royal College of Surgeons in Ireland and University College Dublin Malaysia Campus, 10450 George Town, Malaysia; Upper GI Surgery Unit, Department of Surgery, Hospital Queen Elizabeth I, 88586 Kota Kinabalu, Malaysia; Upper GI Surgery Unit, Department of Surgery, Hospital Queen Elizabeth I, 88586 Kota Kinabalu, Malaysia; Department of Pathology, Hospital Queen Elizabeth I, 88586 Kota Kinabalu, Malaysia; Upper GI Surgery Unit, Department of Surgery, Hospital Queen Elizabeth I, 88586 Kota Kinabalu, Malaysia

**Keywords:** gastric duplication cyst, stomach, gastric mass, laparoscopy, case report

## Abstract

Our paper reports an unusual case of a large gastric duplication cyst (GDC) and a concurrent hepatic cyst in a 50-year-old woman, who presented with severe epigastric pain, weight loss and early satiety. Multimodality imaging was performed before surgery. Laparoscopic resection was favoured but was converted to open due to extensive adhesions. The cyst was completely excised. Post-operative histopathological findings confirmed GDC with no atypia/ malignancy. Our case sheds light on the diagnostic, surgical and post-operative challenges, providing a learning opportunity into this rare entity. Finally, a comprehensive literature review also highlighted the various pathologies that GDC can often mimic.

## Introduction

Duplication cyst are rare developmental anomalies of the alimentary tract, comprising cystic lesions with cellular duplicates of various gastrointestinal structures, mostly diagnosed in childhood [1]. Diagnosis in adults is uncommon and often incidental. Symptoms, if present, are vague GI manifestations such as abdominal pain, bleeding, satiety, or vomiting largely attributed to mass effect and outlet obstruction [2].

Gastric duplication cyst (GDC) is an unusual foregut subtype, representing ~2%–9% of duplications [3]. Typically, GDC are well-circumscribed, non-communicating cystic lesions within the posterior stomach wall [4]. Curative treatment is almost always surgical resection. Though exceedingly rare, studies have reported malignant transformation, primarily adenocarcinoma [4, 5].

Little is currently known about its incidence, aetiology, and malignant potential or long-term outcomes. Diagnostically, GDC often mimics other submucosal pathologies, gastrointestinal stromal tumour (GIST) and pancreatic lesions on imaging, leading to misdiagnosis and treatment delays [3]. This case describes a 50-year-old woman diagnosed with a large GDC and a coexisting hepatic cyst, alongside a literature review highlighting diagnostic challenges of GDC.

## Case

A 50-year-old patient presented with a 2-week history of epigastric and right upper abdominal pain, early satiety and weight loss. Her history included total abdominal hysterectomy with bilateral salpingo-oophorectomy for an ovarian cyst and comorbidities like hypertension, dyslipidaemia, and diabetes mellitus. Examination revealed a firm epigastric mass with mild tenderness extending to right hypochondriac region Hence, US HBS and endoscopy (OGDS) were done to rule out gastric carcinoma.

**Figure 1:**
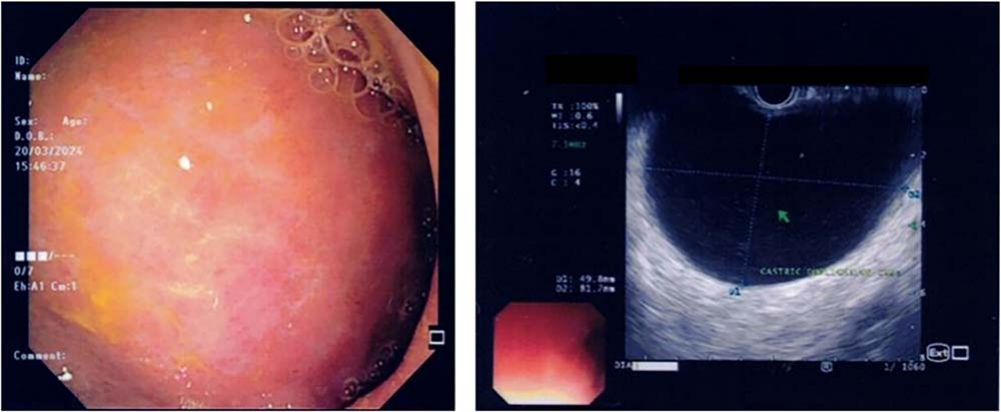
Endoscopic images demonstrate a subepithelial mass externally compressing the antrum. Endosonography revealed a well-defined, homogenous lesion with delineation across multiple gastric wall layers.

**Figure 2:**
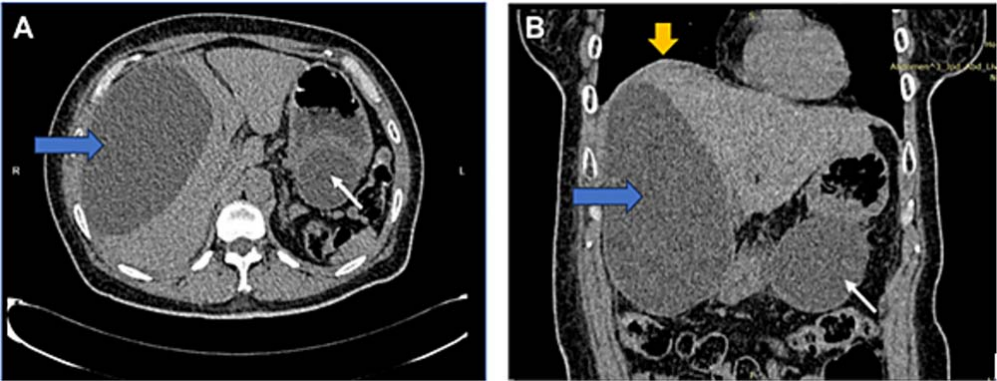
CT image: Axial (A) and coronal (B) view showing a well-defined, homogenous cystic lesion (white arrow) arising from the greater curvature of the stomach. Dimensions were ~ 62/79/147 mm with evidence of external compression to the cardia, fundus, and greater curvature. Another large cystic mass (horizontal arrow) originated from the right liver lobe, measuring 91/146/172 mm. Mass effect was noted with superior displacement of the right hemidiaphragm (vertical arrow). No suspicious or aggressive features of these cystic lesions.

US HBS revealed a cystic lesion in the right liver lobe, raising suspicion for malignancy. OGDS showed a large non-communicating mass, within the distal wall of the greater curvature (Fig. 1). Endoscopic ultrasound (EUS) revealed a well-circumscribed, homogenous stomach cyst (79 × 100 mm). A CT workup was conducted for further clarification, reaffirming the presence of a distinct cystic lesion in the stomach and liver, without signs of malignancy (Fig. 2). At this stage, our findings suggest a large GDC and a coexisting benign hepatic cyst.

**Figure 3:**
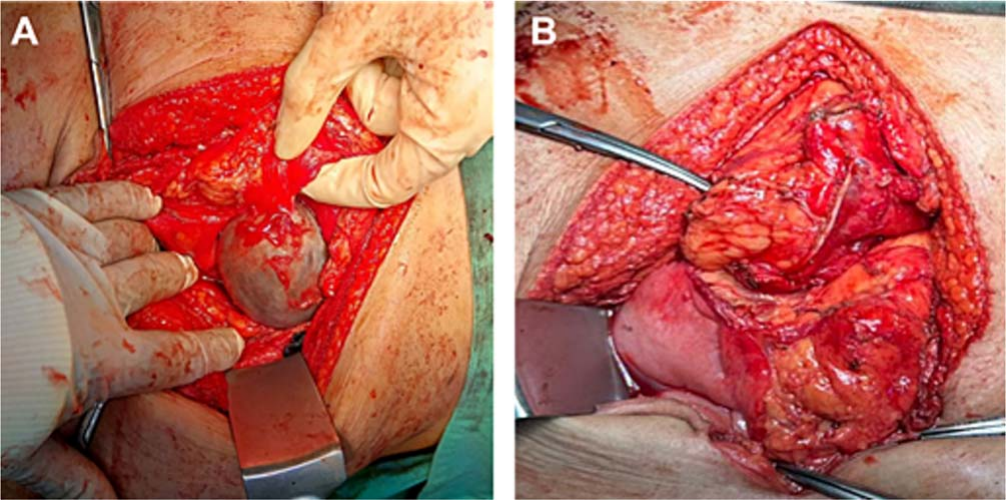
Intraoperative image of GDC before rupturing (A), showing a distinct, well-circumscribed mass measuring ~ 10 × 10 cm with adherence to the posterior stomach wall and tail of the pancreas. Upon excision of the cyst (B) via endo stapler, the staple line along the stomach was further reinforced with sutures.

**Figure 4:**
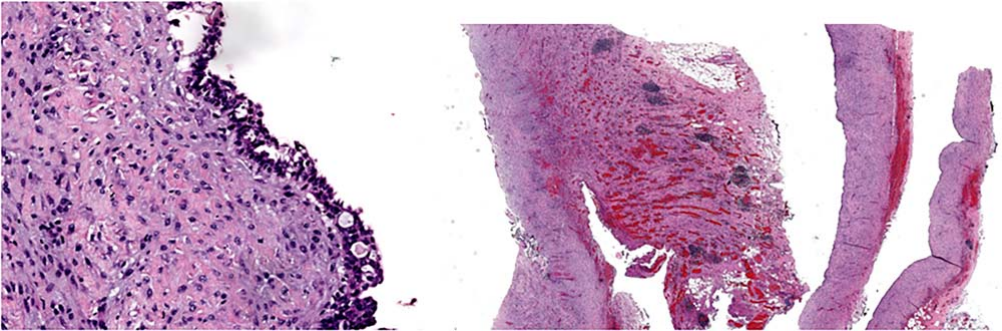
Histology image: Sections show a non-communicating cystic structure with a well-defined smooth muscle layer. No atypia or malignancy. The epithelium is flattened without atypia and occasional mucin-producing cells. Focal granulation tissue seen within smooth muscle bundles.

Give the symptomatology and anatomical impact, GDC resection was prioritized. Planned laparoscopic wedge resection was converted to upper midline laparotomy due to dense adhesions. The GDC, ~10 × 10 cm, originated from the posterior stomach wall with no luminal communication. The cyst ruptured during resection; the stomach wall was reinforced with sutures (Fig. 3). Histopathology confirmed GDC, showing gastric layers and underlying smooth muscle bundles, without atypia (Fig. 4). Post-operatively, the patient developed postprandial vomiting and abdominal pain and were attributed to small bowel adhesions, which resolved with conservative management. The patient achieved full recovery thereafter.

## Discussion

Duplication cysts are rare congenital anomalies, seldom diagnosed in adults [3]. The etiopathology remains unclear, though theories like the aberrant luminal recanalization theory attribute this malformation to errors in first trimester embryogenesis. The dorsal primitive gut, which forms the greater curvature is thought to be the predominant location of GDC [6]. Another plausible explanation is the split notochord theory, where splitting allows GI precursor cells to herniate, forming a duplication cyst [7]. Other mechanisms include intrauterine vascular injuries and anoxia [8, 9].

GDC in adults presents a diagnostic challenge as they mimic pancreatic lesions, pseudocyst, gastric diverticula or GIST on imaging. Confirmation is only via histopathological analysis of the resected tissue, revealing a smooth muscle layer coated with gastric-derived epithelium. Imaging typically involves abdominal CT, showing a hypodense, encapsulated, cystic mass in the upper abdomen [10].

A literature review highlights the diagnostic challenge of GDC (Table 1) due to variations in the anatomic presentation and its implication on the surrounding organs, commonly the pancreas. GDC is often difficult to distinguish from mucinous cystadenomas and other pancreatic neoplasms on CT [10]. Other submucosal growths like GIST are also potential differentials. Few studies elucidated the limited role of magnetic resonance imaging (MRI) in improving diagnostic accuracy [11].

**Table 1:** Studies reporting GDC of the stomach, in adults, mimicking various pathologies

Case	Age/Sex	Presentation	Pre-op diagnosis	Initial diagnosis	Main rationale	Surgery	Location (intra-op)
Caballero 2017	36/F	Abdominal pain, dyspepsia	CT, OGDS, EUS, FNAP	GIST, leiomyoma	Fusiform cells, derived from muscular layer (FNAP)	LG (subtotal), RYGB	Lesser curvature
Deesomsak 2013	52/M	GI bleed, anaemia	OGDS, EUS	Small GIST with cystic changes	Older adult, GI bleed, Umbilicated subepithelial lesion with ulceration (OGDS), atypical findings: central anechoicity, resembles solid rather than cystic (EUS)	Endoscopic submucosal resection	Greater curvature, antral area
Davies 2007	33/M	Epigastric pain	CT	Pancreatic pseudocyst	Close relation to pancreatic head and pylorus (CT)	Exploratory laparotomy	Greater curvature
Maeda 2007	63/F	Asymptomatic	CECT, MRI, EUS, ERCP	Mucinous cystic tumour of pancreas	Close relation to spleen and pancreas (CECT), Cyst wall too thin to appreciate the hypochechoic interlayer (EUS)	Resection and partial gastrectomy	Greater curvature, 4.5 cm
Journo 2004	29/F	Epigastric pain	US, CT, EUS,cyst fluid analysis	Pancreatic mucinous cystadenoma	Young woman, peripheral calcification (CT), high CEA, CA19–9 (aspirate)	Laparotomy, resection	Greater curvature, 8 cm
Johnston 2008	49/F	Asymptomatic	CT, MRI	Cystic neoplasm of pancreas	High CEA, CA19–9 (aspirate), possible involvement of spleen and pancreas (CT)	Exploratory laparotomy	Greater curvature, 4 × 6 × 3 cm
Kavita 2010	42/M	Left lumbar pain	CT	Gastric leiomyoma	Cardia predominance, well-defined density (CT)	Exploratory laparotomy	Lesser curvature

AIHA – autoimmune hemolytic anaemia, CT – computed tomography, ERCP - endoscopic retrograde cholangiopancreaticography, EUS – endoscopic ultrasound, F – female, FNAP – fine needle aspiration puncture, GIST – gastrointestinal stromal tumour, LG – Laparoscopic gastrectomy, M – male, OGDS – esophagogastroduodenoscopy, RYGB – Roux-en-Y gastric bypass.

EUS has emerged as a promising adjunctive modality, offering enhance characterization, distinguishing GIST and other malignancies [12]. In our patient with GDC and a hepatic cyst, EUS offered greater diagnostic confidence with clearer delineation of subepithelial features, and dimensions. However, atypical EUS findings had led to misdiagnosis in some reports (Table 1) [10, 13]. EUS may not always depict GDC accurately and thus must remain a candidate for differential diagnosis.

EUS also allows for endoscopic FNA and fluid analysis. Though high concentrations of CEA and CA19-9 may suggest malignant pancreatic lesions, previous cases show that the relationship of tumour markers is often complex (Table 1). In our patient, FNA was avoided to prevent unnecessary risk of infection, bleeding, and tumour seeding.

There is no standardised management algorithm to date. Large symptomatic GDC is generally treated by resection. Benefits of surgery include curation, histopathological confirmation, and prevention of malignancy. Prognosis is generally good, with low complication rates [14]. Our patient recovered well despite transient adhesion obstruction. Other cases have reported post-operative complications such as GERD, esophagitis, or Barrett's esophagus [14].

Given the diagnostic difficulty, there are concerns of misdiagnosis complicating surgical intervention, In Cabellero *et al.*, laparoscopic gastrectomy was performed due to GDC being misdiagnosed as GIST (Table 1). Some practitioners also advocate for expectant management in asymptomatic patients [14]. However, rare cases of malignant transformation in to adenocarcinoma have been documented, potentially favouring early surgical excision [4, 5]. A review by Abdulla *et al.* reported only 11 such cases [15]. Prognosis in these cases is often poor, with high metastatic rates.

Although our specimen lacked well-defined gastric glands, histopathology supported a diagnosis of GDC. Several papers have also elucidated the histological heterogeneity of GDC [2]. The epithelial flattening and granulation are likely reflecting chronic inflammatory reactions.

Due to its rarity, the literature remains scarce and primarily of low-quality evidence. More studies are needed to clarify long-term outcomes, malignant potential and recurrence, and to drive efforts to standardize treatment algorithms of GDC. Research on embryogenesis may also reveal on specific genetic and syndromic association to GDC. Advances in robotic surgery and imaging technology like EUS offers greater potential for improved patient outcomes.

## Conclusion

This case entails a rare presentation of GDC in an adult patient. The case highlights critical diagnostic challenges and the pivotal role of multimodal imaging. A concurrent hepatic cyst further complicated the diagnosis. Despite planned laparoscopy, surgery was converted to open due to dense adhesions. Full recovery was achieved thereafter.
